# Dietary crude glycerol as an energy source in broiler chickens: a meta-analysis

**DOI:** 10.5713/ab.250686

**Published:** 2026-03-11

**Authors:** Rasheed Olayiwola Sulaimon, Ridwan Olalekan Oyeniyi, Yusup Sopian, Katatikarn Sahatsanon, Anuraga Jayanegara, Panneepa Sivapirunthep, Pattraphorn Patthararangsarith, Chanporn Chaosap

**Affiliations:** 1 Doctoral Program in Innovative Tropical Agriculture, School of Industrial Education and Technology, King Mongkut’s Institute of Technology Ladkrabang, Bangkok, Thailand; 2 Department of Animal Science, Faculty of Agriculture, University of Abuja, Abuja, Nigeria; 3 Department of Animal Production, Faculty of Agriculture, University of Ilorin, Ilorin, Nigeria; 4 Department of Animal Nutrition and Feed Technology, Faculty of Animal Science, IPB University, Bogor, Indonesia; 5 Department of Agricultural Education, School of Industrial Education and Technology, King Mongkut’s Institute of Technology Ladkrabang, Bangkok, Thailand

**Keywords:** Broiler Chickens, Crude Glycerol, Meta-analysis, Performance, Rearing Phase, Strain

## Abstract

**Objective:**

This meta-analysis evaluated the effects of dietary crude glycerol (CG) supplementation on growth performance, carcass traits, meat quality, and blood biochemical parameters in broiler chickens.

**Methods:**

A systematic search of peer-reviewed studies in Scopus, ScienceDirect, and Google Scholar was performed following the Preferred Reporting Items for Systematic Reviews and Meta-Analyses protocol. Twenty-five eligible studies were included. Effect sizes were calculated as standardized mean differences using a random-effects model in OpenMEE software. Subgroup analyses were performed according to strain, sex, rearing phase, and inclusion level.

**Results:**

CG supplementation increased body weight gain (p<0.05) and improved feed conversion ratio (p<0.05), while feed intake was unaffected. Among carcass traits, breast yield increased (p<0.05), whereas carcass yield and thigh yield were not affected. CG inclusion also reduced meat ash content and ultimate pH, while drip loss increased, indicating potential negative effects on water-holding capacity. Blood biochemical indices, including reduced alanine aminotransferase and elevated alkaline phosphatase, indicated adaptive physiological responses without evidence of toxicity. Subgroup analyses revealed that strain, sex, and inclusion level moderated growth outcomes (p<0.05).

**Conclusion:**

CG can be incorporated into broiler diets as an alternative energy source to enhance growth performance without compromising carcass yield. However, its influence on meat quality highlights the need to optimize inclusion levels and consider variability among glycerol by-products before large-scale application.

## INTRODUCTION

The global poultry industry, a leading contributor to animal protein production, is under increasing pressure to enhance productivity while maintaining cost-efficiency and sustainability. Feed cost represents approximately 60% to 70% of the total expenses in poultry production, with energy-rich ingredients like corn constituting the major share [1]. However, the growing competition between humans and animals for cereal grains, fluctuating market prices, and regional scarcity have necessitated the search for alternative, cost-effective, and sustainable energy sources. Among the agro-industrial by-products, crude glycerol (CG), a co-product from biodiesel production, has gained considerable attention as a promising energy-rich feed ingredient for broiler diets [2–4].

CG is primarily composed of glycerol (70% to 80%), along with water, methanol, salts, fatty acids, and other residues depending on its source and production method [5,6]. With the rapid global expansion of biodiesel production, particularly in countries facing energy insecurity or promoting biofuel programs, the generation of CG has increased exponentially [7,8]. For every 1,000 kg of biodiesel produced, approximately 100 kg of CG is generated [9]. This surplus often exceeds industrial demand, posing both economic and environmental disposal concerns [10]. Consequently, the valorization of CG in animal feed has become an attractive and practical avenue for mitigating waste while reducing feeding costs. The energy content of CG ranges from 3,250 to 4,134 kcal/kg, making it comparable to conventional ingredients like corn and soybean oil [4,8,11]. Glycerol, the main component of CG, is readily absorbed in the gastrointestinal tract and can be metabolized via glycolysis or gluconeogenesis to produce energy [12,13]. Several studies have demonstrated that CG can replace a portion of dietary corn without compromising broiler growth performance, feed conversion ratio (FCR), or carcass yield [14–16]. In fact, CG has also been linked to improvements in pellet quality due to its hygroscopic and binding properties [4].

Despite its potential, findings on CG inclusion in broiler diets remain inconsistent. While some researchers reported no adverse effects at inclusion levels up to 5% to 7.5% [16,17], others observed metabolic alterations such as increased blood glucose, water intake, or fecal moisture at higher inclusion levels [16,18]. The variability in CG’s nutritive value and broiler response is attributed to differences in CG purity, source, processing method, and inclusion level [4,19]. For example, CG sourced from soybean oil, palm oil, or animal fats may differ in glycerol concentration, residual methanol content, and associated impurities, which can significantly influence animal health and performance [3,20]. Furthermore, some studies highlighted reductions in breast yield [21] and inconsistent effects on gut morphology and blood biochemistry [16,19], whereas others found no significant changes in carcass traits [22].

Given the growing interest in CG as a dietary energy source and the lack of consensus in the literature, there is a compelling need for an evidence-based synthesis of its effects across multiple biological and productive parameters in broiler chickens. To date, no meta-analysis has systematically integrated the diverse and fragmented findings on CG supplementation in poultry diets. Meta-analysis serves as a powerful statistical tool to consolidate quantitative data from multiple studies, evaluate heterogeneity, assess dose-response relationships, and generate more reliable conclusions than individual experiments alone [23].

Therefore, the objective of this meta-analysis was to quantitatively evaluate the effects of CG inclusion in broiler diets on growth performance, carcass traits, meat quality, and blood biochemical parameters. This synthesis may provide practical guidance to poultry nutritionists, feed formulators, and industry stakeholders on the optimal utilization of CG as an alternative energy source, contributing to cost reduction and sustainability in broiler production.

## MATERIALS AND METHODS

### Literature search and study selection

A systematic search was conducted to identify peer-reviewed studies on the effects of dietary CG as an alternative energy source in broiler diets, following the Preferred Reporting Items for Systematic Reviews and Meta-Analyses (PRISMA) guidelines. Scopus, ScienceDirect, and Google Scholar were queried using the keywords “glycerol,” “glycerine,” “crude glycerol,” “crude glycerine,” “broiler,” “performance,” “meat quality,” and “blood parameters,” combined with Boolean operators (AND/OR) and without year restrictions. Reference lists of relevant articles were manually screened to capture additional eligible studies not retrieved through the database search. Study selection followed the PICO framework: Population (broilers), Intervention (CG inclusion), Comparison (basal diets), and Outcomes (growth performance, carcass traits, meat quality, and blood biochemistry), consistent with recent poultry nutrition meta-analyses [23,24].

### Inclusion and exclusion criteria

**Figure 1:**
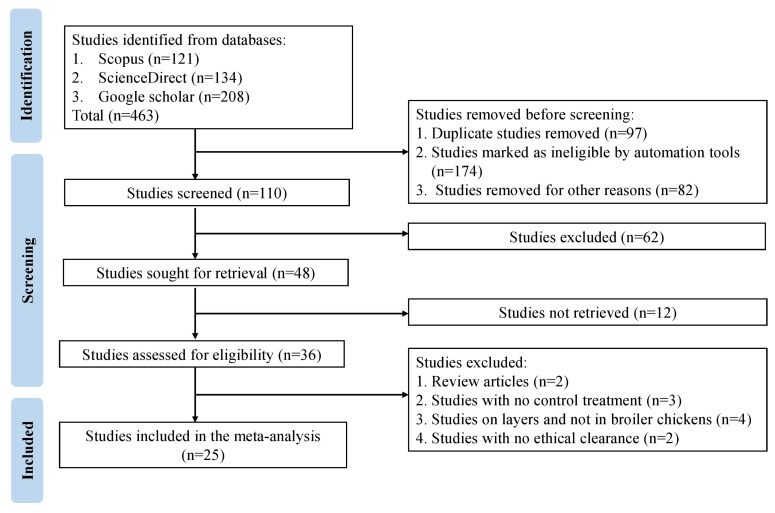
Literature search and selection process following the PRISMA procedure. PRISMA, Preferred Reporting Items for Systematic Reviews and Meta-Analyses.

Studies were screened using the following inclusion criteria: (i) trials involving healthy commercial broiler chickens with reported ethical approval; (ii) original articles published in English that reported at least one relevant outcome (growth performance, carcass traits, meat quality, or blood biochemistry) with data expressed as means and measures of variance (standard deviation [SD] or standard error of the mean [SEM]) along with sample size; (iii) clear description of the type and inclusion level of CG; and (iv) the presence of a control group without CG supplementation. Exclusion criteria were: (i) studies lacking a control group; (ii) grey literature, reviews, or unpublished theses; (iii) trials where CG was combined with other additives; (iv) studies deviating from standard broiler feeding practices; and (v) studies without ethical approval. Following this process, 463 articles were initially identified, of which 25 met all criteria and were included in the meta-analysis. The study selection process is illustrated in the PRISMA flowchart (Figure 1).

### Data extraction

From each eligible study, the following details were recorded: first author’s surname, year of publication, study location (country and continent), broiler strain, sex, rearing phase, CG inclusion level, trial duration, number of birds, and number of treatments. Outcome data for control and CG groups included means, measures of variance (SD or SEM), and sample sizes. When SEM was reported, it was converted to SD using the formula: $SD=SEM\times \sqrt{n}$, [25]. The CG inclusion expressed in g/kg was converted to percentage (%) by dividing by 10. In studies with multiple inclusion levels, each CG treatment was compared with the control. All extracted datasets from the 25 included studies were compiled and formatted into comma-separated values (CSV) files using Microsoft Excel 2021, compatible with the OpenMEE software applied in this meta-analysis.

### Data analysis

The meta-analysis was conducted using a random-effects model in the OpenMEE software [26], applying the DerSimonian-Laird method [27]. Effect sizes were expressed as standardized mean differences (SMDs) with 95% confidence intervals (CI), following the guidelines of Koricheva et al [28]. A significance level of 5% was applied for all pooled estimates. Hedges’*d* was used to calculate SMDs, which accounts for small sample size bias, and effect sizes were computed for each comparison between treatment and control groups as described by Sopian et al [29]. Between-study variance was estimated using the DerSimonian-Laird method, and random-effects models were applied to allow for expected variability across studies arising from differences in broiler strain, diet formulation, and trial conditions.

#### Heterogeneity assessment

Variability among studies was quantified using the *I^2^* statistic, which represents the percentage of total variation across studies that is attributable to heterogeneity rather than sampling error [25]. The degree of heterogeneity was categorized as follows: insignificant (0<*I^2^≤* 25%), low (25<*I^2^≤*50%), moderate (50<*I^2^≤*75%), and substantial (*I^2^*>75%) [30]. Where substantial heterogeneity was detected, additional analyses such as subgrouping were performed to identify potential sources of variation.

#### Subgroup and publication bias analyses

**Figure 2:**
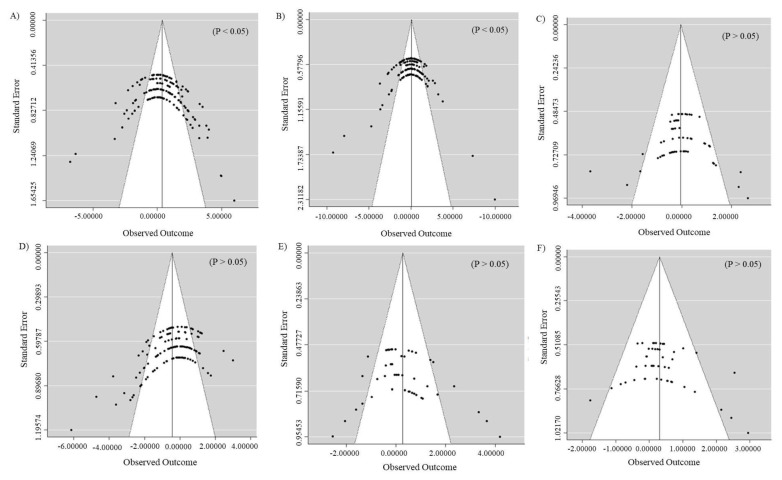
Funnel plots illustrating the effects of dietary crude glycerol on (A) BWG, (B) FCR, (C) FI, (D) carcass yield, (E) breast, and (F) thigh in broilers. BWG, body weight gain; FCR, feed conversion ratio; FI, feed intake.

Subgroup analyses were conducted to explore the effect of study-level factors on outcomes. Moderators included (i) broiler strain (Cobb 500 and Ross 308), (ii) sex (male, mixed groups), (iii) rearing phase was defined by age (days), for growth performance, phases included starter (1 to 14 d), grower (14 to 28 d), finisher (28 to 42 d), and the overall period (1 to 42 d), while carcass traits were evaluated at standard slaughter ages of 21 d (starter) and 42 d (finisher), and (iv) CG inclusion level, classified as low (1% to 3%), low-to-moderate (>3% to 7%), moderate (>7% to 12%), and high (>12% to 21%). These categories reflected the ranges used in eligible studies. Analyses were restricted to parameters with ≥45 observations and ≥5 comparisons, prioritizing groups with the largest sample sizes. Performance traits including body weight gain (BWG), feed intake (FI), and FCR and carcass traits (carcass yield, breast yield, and thigh yield) were examined. Publication bias was assessed using funnel plots (Figure 2), and Egger’s test was performed in MetaWin 3 software [31], with significance at p<0.05 [32]. When bias was evident, sensitivity analysis was applied by sequentially excluding individual studies to determine their effect on pooled estimates, thereby confirming the stability of the results under high heterogeneity [30].

## RESULTS

### Characteristics of studies used in the meta-analysis

A total of 463 studies were identified in various search engines, of which 25 were eligible and selected for the meta-analysis (Table 1). The eligible articles revealed that most of the studies were published between 2006 and 2024 from four continents (Asia, Europe, South and North America) in nine different countries (USA, Greece, Turkey, Canada, Brazil, Thailand, Spain, Iraq, and India). A total of 19,511 male and mixed-sex broilers from Ross 308 and Cobb 500 strains aged between 1 and 42 days were used, and CG was used as a partial replacement of corn at inclusion levels ranging from 0% to 21% in studies with treatment groups ranging from 2 to 6, and control as one of the treatments. All the 25 selected studies were evaluated for growth performance variables, carcass traits, meat quality parameters, and blood biochemicals. Our findings revealed that nearly half of the studies included in this meta-analysis were conducted in South America, with Brazil accounting for 12 out of 25 studies (48%). This geographic representation aligns with Brazil’s leading role in the global poultry sector; in 2024, Brazil produced approximately 14.6% of the world’s chicken meat [33].

### Growth performance

The meta-analysis revealed that dietary inclusion of CG significantly affected key performance metrics in broiler chickens (Table 2). Specifically, BWG increased with a SMD of 0.398 (95% CI: 0.203 to 0.592; p<0.001). Similarly, FCR was reduced (SMD = −0.444; 95% CI: −0.616 to −0.272; p<0.001), and by contrast, FI was not affected by CG supplementation (SMD = 0.061; p = 0.481). Despite these significant effects, moderate to substantial heterogeneity was observed across studies (I^2^ = 74.2% for BWG, 65.4% for FCR, and 68.1% for FI, all with p<0.001), indicating variability in study outcomes.

### Carcass traits

The CG supplementation had selective effects on carcass traits (Table 3). Breast yield was increased (SMD = 0.307; 95% CI: 0.116 to 0.498; p = 0.002), as well as drumstick yield (SMD = 0.350; 95% CI: 0.016 to 0.683; p = 0.040). Back yield also showed a positive response (SMD = 0.769; 95% CI: 0.283 to 1.254; p = 0.002). In contrast, abdominal fat was reduced (SMD = −0.368; p = 0.130, not statistically significant at 5%, though the negative effect is notable), while carcass yield, thigh yield, and wings were not affected by CG. For internal organ weights, liver weight was significantly increased (SMD = 0.387; 95% CI: 0.008 to 0.766; p = 0.045), whereas proventriculus weight was reduced (SMD = −0.594; 95% CI: −1.010 to −0.178; p = 0.005). No significant effects were observed in gizzard, heart, pancreas, or spleen weights due to CG supplementation. Heterogeneity was moderate to high for most carcass and organ traits (I^2^ ranging from 36.1% to 74.8%), suggesting variability among studies, particularly for drumstick, thigh, and liver weights.

### Meat quality parameters

The analysis of meat quality traits showed that dietary CG had varied effects (Table 4). Ash content was significantly reduced (SMD = −1.061; 95% CI: −1.648 to −0.473; p<0.001), among physical quality indicators, meat pH was decreased (SMD = −0.326; p = 0.042), while drip loss was increased (SMD = 0.582; p = 0.004), suggesting a decline in water-holding capacity. Other measured traits, including ether extract, crude protein, moisture, cook loss, shear force, and color indices (L*, a*, b*), were not significantly influenced by CG supplementation. However, some of these non-significant outcomes still exhibited moderate-to-high heterogeneity, ether extract I^2^ = 70.2%, moisture I^2^ = 55.6%, and yellowness I^2^ = 74.2%, reflecting variability in individual study results. Notably, heterogeneity for significant outcomes like drip loss and pH was low (I^2^ = 24.3% and 17.2%, respectively), strengthening the reliability of these findings.

### Blood biochemical parameters

The CG supplementation produced several significant effects on blood metabolites (Table 5). Cholesterol was reduced (SMD = −0.743; 95% CI: −1.187 to −0.299; p = 0.001), as were albumin (SMD = −1.388; p = 0.002), ALT (SMD = −1.000; p = 0.006), and low-density lipoprotein (LDL) (SMD = −1.317; p<0.001). Conversely, glucose levels were elevated (SMD = 1.184; p = 0.002), as were ALP (SMD = 0.530; p = 0.031) and high-density lipoprotein (HDL) (SMD = 0.665; p = 0.001). Other parameters, including triglycerides, total protein, creatinine, globulin, and AST, did not show significant differences between CG-fed and control broilers. The heterogeneity analysis indicated considerable variability for certain traits, such as creatinine (I^2^ = 85.7%), albumin (I^2^ = 73.2%), and AST (I^2^ = 69.0%). However, for some significant outcomes, heterogeneity was low in HDL (I^2^ = 3.1% and LDL I^2^ = 8.9%), which strengthens the robustness of these findings.

### Subgroup analysis on the effect of dietary crude glycerol on growth performance in broiler chickens

Subgroup analysis revealed that strain, sex, rearing phase, and inclusion level of CG significantly influenced growth performance outcomes in broiler chickens (Table 6). For BWG, Ross 308 demonstrated a higher response to CG inclusion (SMD = 0.905, p<0.001) compared with Cobb 500, which showed no significant effect. Male broilers exhibited greater BWG (SMD = 0.345, p = 0.001), and mixed-sex groups also responded positively (SMD = 0.747, p = 0.003). Regarding rearing phases, significant improvements were observed during the starter (SMD = 0.571, p = 0.021) and grower phases (SMD = 0.808, p = 0.009), whereas the finisher phase did not yield a notable effect. At the level of inclusion, a clear trend was observed, as low-to-moderate inclusion levels (>3% to 7%) enhanced BWG (SMD = 0.973, p<0.001), while high inclusion levels (>12% to 21%) impaired BWG (SMD = −0.747, p = 0.024). For FI, Ross 308 broilers showed a reduction (SMD = −0.663, p = 0.008), while males (SMD = −0.447, p<0.001) and mixed-sex groups (SMD = −0.471, p = 0.010) also demonstrated reduced intake compared with controls. Interestingly, during the grower phase, FI was increased (SMD = 0.504, p = 0.035), whereas other phases remained unaffected. Similar to BWG, low-to-moderate glycerol inclusion levels (>3% to 7%) were associated with increased FI (SMD = 0.507, p<0.001). In terms of FCR, Ross 308 again showed an improvement (SMD = 0.540, p<0.001), whereas Cobb 500 and sex subgroups had no effect. During the starter phase, CG supplementation improved FCR (SMD = −0.673, p<0.001), but no effects were observed in later phases. With respect to inclusion level, both very low (1% to 3%) (SMD = −0.326, p = 0.012) and low-to-moderate (>3% to 7%) levels (SMD = −0.716, p<0.001) improved FCR, whereas high inclusion levels (>12% to 21%) worsened FCR (SMD = 0.554, p = 0.014).

### Subgroup analysis on the effect of dietary crude glycerol on carcass traits in broiler chickens

Subgroup analysis revealed that certain moderators had a significant influence on carcass traits in broilers supplemented with CG (Table 7). For carcass yield, the effect was not significant across most subgroups, except in the starter phase (21 d), where a positive response was observed (SMD = 1.087, p = 0.058), although this effect was marginal and not consistently reflected in the overall rearing period. Regarding breast yield, strain influenced outcomes, with Cobb 500 exhibiting a higher response (SMD = 0.302, p = 0.004), while Ross 308 showed no significant effect. Male broilers demonstrated an increase in breast yield (SMD = 0.333, p = 0.005). The rearing phase also played a role, as supplementation during the starter phase (21 d) significantly improved breast yield (SMD = 1.161, p = 0.012), and a significant but smaller effect was retained when considered across the full rearing period (42 d; SMD = 0.251, p = 0.008). In terms of inclusion levels, low-to-moderate supplementation (>3% to 7%) positively affected breast yield (SMD = 0.326, p = 0.015). For thigh yield, significant moderators were pronounced in the rearing phase and inclusion level. A beneficial effect was evident across the entire rearing period (42 d; SMD = 0.392, p = 0.016). Furthermore, high inclusion levels of CG (>12% to 21%) enhanced thigh yield (SMD = 1.091, p = 0.010), while lower inclusion levels did not demonstrate a consistent effect.

## DISCUSSION

### Growth performance

Our meta-analysis revealed that dietary inclusion of CG produced a clear and consistent improvement in growth efficiency in broiler chickens. The pooled results showed a significant increase in BWG, and the FCR was reduced, indicating better feed efficiency in birds receiving CG. In contrast, FI remained essentially unchanged. Practically, these findings suggest that CG can serve as an adequate partial replacement for conventional energy ingredients like corn, without compromising intake levels. The improvement in BWG and FCR observed in this meta-analysis can be explained by the efficient utilization of CG as a complementary energy source rather than a superior substitute for corn. Although corn starch remains the primary and most efficient source of dietary glucose in broiler nutrition, CG provides a highly digestible energy fraction, with a gross energy value of approximately 3,600 to 3,700 kcal/kg, comparable to that of corn [8]. Glycerol is rapidly absorbed in the small intestine and enters hepatic metabolism as a gluconeogenic precursor, contributing to circulating glucose and sparing dietary starch for other metabolic functions [8,9,34]. Importantly, this pathway does not replace direct glucose absorption from corn-derived starch but complements it, potentially smoothing postprandial energy availability and reducing metabolic energy losses [4]. Such complementary energy utilization may explain the consistent improvements in feed conversion efficiency and BWG observed across studies when CG was partially replaced by corn. The neutral effect on FI indicates that CG does not alter palatability or satiety when incorporated within practical inclusion levels but rather enhances nutrient utilization efficiency, explaining the better FCR observed [4]. When comparing our pooled findings with experimental studies, there is good concordance with several original trials. Improving growth and feed efficiency in birds fed vegetable-derived glycerin, supporting the positive BWG and FCR effects observed in the meta-analysis [35]. Topal and Ozdogan [36] similarly reported improved BWG with dietary CG inclusion, especially at the higher tested inclusion levels, achieving an acceptable performance and carcass yield across a range of inclusion levels, which reinforces the practical viability of CG as an energy substitute [4,37]. The modest inclusion levels observed during starter phases can be particularly beneficial, consistent with the notion that age and rearing phase moderate the response [1,38]. Conversely, some studies report neutral or dose-dependent results. For instance, Silva et al [39] found no clear advantage of specific purified glycerin formulations in certain performance metrics, and Romano et al [16] highlighted metabolic adjustments that occurred when glycerol was increased, indicating potential limits to beneficial inclusion rates. Review syntheses summarize this mixed but generally favorable body of evidence and emphasize that effects are contingent on glycerol quality and dietary context [8].

### Carcass traits

The positive shifts in breast, drumstick, and back yields indicate that CG can favorably alter carcass composition by promoting lean-tissue accretion in key commercial cuts. Such repartitioning toward breast and leg muscles is desirable commercially because it raises the value of carcass output per bird even when overall carcass yield remains similar [40]. Glycerol is an energetic, gluconeogenic precursor that can rapidly supply metabolizable energy to support protein synthesis and muscle accretion; when CG replaces part of cereal energy, the altered energy balance and greater readily available substrate may encourage lean growth in breast and leg muscles [16,22,35]. The observed liver enlargement is consistent with increased hepatic metabolism of glycerol, such as gluconeogenesis, lipogenesis, and related pathways, as the liver is the major site for glycerol conversion to glucose or glycerol-3-phosphate. Hepatic hypertrophy or increased metabolic enzyme induction is a commonly reported response when dietary CG is included [4,16]. Conversely, reductions in gizzard and proventriculus weights likely reflect dietary physical characteristics. CG inclusion frequently reduces dietary particle size or fiber content and increases dietary solubility, which can lower the grinding and muscular workload of these digestive organs and lead to smaller organ mass [36,37]. Several primary studies report similar patterns. Urgnani et al [35] and Broch et al [37] observed maintenance or modest improvements in breast and leg yields at practical CG inclusion levels, supporting our pooled positive effects on commercial cuts. Topal and Ozdogan [36] and Romano et al [16] described hepatic responses and changes in internal-organ weights when glycerol was fed, consistent with the increased liver size in the meta-analysis. However, effects on overall carcass yield are inconsistent across trials and were non-significant in the pooled result. This is likely because small increases in specific cuts may be offset by stable or slightly reduced yields in other parts, resulting in net-neutral carcass percentages in some studies [4,39]. The substantial heterogeneity (I^2^) for several traits suggests the magnitude of these effects depends on factors such as glycerol purity, inclusion rate, diet formulation (what fraction of maize or oil is replaced), bird strain or age, and trial management, factors previously identified as moderators in the literature [1,8].

### Meat quality

The observed reduction in ash content in meat from CG-fed broilers compared with controls suggests that replacing maize with CG may slightly dilute mineral deposition in muscle tissue. This could be due to differences in mineral composition between maize and CG, as CG is primarily an energy source with limited mineral contribution [41]. Similar reductions in ash deposition have been reported in carcass studies where dietary substitution with energy-dense but mineral-poor ingredients lowered mineral accretion in muscle [42,43]. Similarly, muscle pH was significantly reduced in broilers receiving CG diets. Lower ultimate pH indicates greater post-mortem glycolysis, which may be linked to shifts in carbohydrate availability and glycogen storage dynamics when CG replaces part of the maize fraction. Romano et al [16] suggested that dietary glycerol, being rapidly metabolized, could alter glycogen reserves, leading to more lactic acid accumulation and thus a lower pH at rigor mortis. Although the reduction was modest, this shift could have downstream implications for meat color and water retention. In contrast, drip loss increased significantly with CG supplementation. The rise in drip loss may be explained by the reduced pH, as lower ultimate pH decreases the net charge of muscle proteins, reducing their ability to bind water and thereby increasing exudate during storage. Such a combination of lower pH and higher drip loss in broiler meat may be attributed to alterations in water distribution within the muscle. A rapid decline in pH post rigor reduces the water-holding capacity of myofibrillar proteins, leading to a shift from immobilized water to free water [44,45]. This increased proportion of free water facilitates its release from the muscle structure, thereby accounting for the higher drip loss observed. In this study, subgroup analysis was not performed for meat quality parameters due to the limited number of eligible studies reporting these outcomes. Consequently, definitive inclusion thresholds could not be statistically established. Nevertheless, qualitative examination of the four studies [21,22,35,39] that reported meat quality traits revealed a consistent pattern in which low to moderate CG inclusion levels (generally>3%–7% of the diet) did not adversely affect meat pH or drip loss, whereas higher inclusion levels were more frequently associated with reductions in pH and increased exudative losses. This observation aligns with the findings of Naitam et al [46], who reported elevated drip loss in broilers fed CG-based diets, and Carvalho et al [40], who suggested that excessive inclusion of glycerin can compromise meat water-holding capacity. Increased drip loss has practical implications, as it may reduce juiciness and negatively affect consumer acceptability, even if overall growth performance remains unaffected. On the other hand, meat color (L*, a*, and b*) and texture traits such as shear force were not significantly altered. This indicates that CG inclusion does not affect myoglobin oxidation or muscle fiber structural integrity, preserving visual quality and tenderness. These neutral effects are consistent with the findings of Silva et al [39] and Urgnani et al [35], who also reported no differences in color or tenderness of broiler breast meat when glycerol replaced part of the energy in diets.

### Blood biochemical parameters

The rise in circulating glucose is a predictable metabolic consequence of dietary CG, which is efficiently absorbed from the intestine and converted in the liver to glucose via gluconeogenesis; this raises blood glucose availability and explains the strong pooled response [4,16]. Changes in lipid-related markers like lower total cholesterol and LDL, and higher HDL, suggest that glycerol feeding may beneficially modulate lipid metabolism and lipoprotein partitioning. Possible mechanisms include altered hepatic lipogenesis and VLDL synthesis (glycerol provides glycerol-3-phosphate for triglyceride backbone formation but may also shift lipid export patterns), improved fatty-acid oxidation, or changes in bile acid metabolism; such effects have been reported in individual trials and reviews noting favorable cholesterol responses with glycerol inclusion [8,35]. The LDL reduction and HDL increase indicate a potentially cardioprotective serum profile in birds, which is important for metabolic health. The decrease in serum albumin and in ALT with a small increase in ALP suggests some hepatic functional shifts rather than frank hepatic damage. ALT is commonly used as a marker for hepatic injury when elevated; its observed reduction in our meta-analysis under CG feeding likely does not indicate liver pathology but may reflect altered enzyme kinetics, sampling timing, or metabolic adaptation [47]. Meanwhile, the elevated ALP may be more indicative of active bone mineralization or bile flow, rather than hepatic dysfunction, given ALP’s established role as a marker for bone formation in growing birds [48]. The observed increase in liver weight, combined with these enzyme changes, supports the idea of increased hepatic metabolic activity rather than pathology [16,36]. Several studies in our dataset corroborate these directions. Urgnani et al [35] and Broch et al [37] reported lower serum cholesterol with glycerin inclusion, and de Castro Tavernari et al [4] documented changes in metabolizable energy and blood metabolites consistent with higher blood glucose when glycerin is fed. Conversely, some trials show minimal or inconsistent biochemical changes depending on glycerol purity and formulation [9,39]. Ghayas et al [8] summarize that lipid-profile benefits are commonly observed but are sensitive to diet context and glycerol characteristics.

### Subgroup analysis

#### Strain

The subgroup analysis demonstrated that strain significantly influenced the responses of broilers to supplementation with CG. Ross 308 broilers exhibited improved BWG and FCR, alongside reduced FI, whereas Cobb 500 showed limited responsiveness. This finding may be attributed to inherent genetic differences in growth potential, nutrient partitioning, and feed efficiency between the two strains. Ross broilers are generally characterized by superior feed efficiency and adaptability to dietary energy density, which may explain their favorable performance when CG was included as an alternative energy source. Similar strain-specific effects were reported by Awad et al [49], who observed differential performance responses between Ross 308 and Cobb 500 under varying dietary and environmental conditions, confirming that genetic background modulates nutrient utilization efficiency. Similar trends were also noted by Manyeula et al [50] and Ogbuewu et al [51], who observed variations in FI among broiler strains offered comparable dietary treatments.

Carcass traits revealed important differences across moderators. Considering strain, breast yield was significantly higher in Cobb 500 compared with Ross 308, whereas carcass yield and thigh yield showed less variation. The superiority of Cobb in breast yield can be attributed to genetic selection, as Cobb lines have been strongly selected for breast muscle accretion, leading to higher carcass efficiency and lean yield. This genetic predisposition enables Cobb birds to utilize additional energy substrates such as glycerol more effectively for lean deposition. In this context, CG utilization in Ross 308 appears to preferentially support metabolic efficiency and overall lean growth rather than muscle deposition. The improved BWG and FCR observed in Ross 308 suggest that dietary CG was primarily directed toward meeting energy demands and enhancing feed efficiency, whereas muscle accretion, particularly in breast muscle, remained genetically constrained. Awad et al [49] similarly reported that Cobb broilers outperformed Ross in breast development under nutritional interventions, supporting the present findings. By contrast, thigh yield remained relatively unaffected by strain, which could be due to the fact that thigh muscle growth is less influenced by genetic selection for carcass traits and more dependent on general nutrient supply.

#### Sex

Sex also emerged as a significant moderator, with male and mixed-sex groups fed CG diets showed improvements in BWG and reductions in FI compared with control groups, highlighting the efficiency of glycerol as an energy source. Interestingly, the mixed-sex groups showed a slightly higher SMD than males, suggesting that the presence of both sexes may enhance the overall flock response to CG. This may reflect compensatory growth dynamics, where males drive rapid lean tissue accretion while females contribute to more efficient nutrient partitioning. These findings align with the broader evidence that sex influences nutrient utilization, with males generally demonstrating greater lean tissue growth potential, yet flock composition (mixed vs. single sex) can influence feed efficiency outcomes under different energy densities [52]. Recent work has similarly shown that sex-specific physiology and nutrient metabolism affect growth performance and feed efficiency in broilers, with males typically outperforming females but mixed-sex rearing providing balanced flock-level responses [53].

When sex was considered, no significant differences were observed for carcass yield, breast yield, or thigh yield. This absence of effect suggests that CG supplementation may have provided similar energy utilization across both sexes, minimizing expected differences. Typically, males grow faster and deposit more lean tissue than females, but the lack of significance here indicates that the magnitude of glycerol’s effect was not strong enough to interact with sex-driven differences in nutrient partitioning. Ukwu et al [54] noted that while sex influences metabolic rate and carcass composition, nutritional interventions sometimes fail to elicit sex-specific effects, which is consistent with the present outcome.

#### Rearing phase

Rearing phase further influenced the response to CG inclusion, with significant improvements observed in the starter and grower phases for BWG and FI, respectively, while some notable effects were recorded in the finisher period. The benefits in early growth phases may be attributed to the rapid intestinal development and high energy demand of young broilers, which enhance the utilization of glycerol as a gluconeogenic substrate. Cerrate et al [55] similarly reported that CG inclusion during the starter phase supported BWG and FCR, with diminishing responses in later stages when growth rate begins to plateau, and fat deposition predominates.

At the rearing phase, subgroup analysis of carcass traits was limited to the starter (21 d) and finisher (42 d) phases, consistent with the slaughter points reported in the included studies. Carcass yield and breast yield were more responsive during the starter phase, whereas thigh yield tended to improve in later phases. These observations were based on carcass evaluations conducted at specific ages corresponding to each of the two feeding phases. The enhanced carcass and breast yield during the starter phase may be explained by the high rate of muscle fiber hypertrophy and protein accretion during early growth [56], processes that benefit from rapidly available energy sources such as glycerol. Boonwong et al [21] reported similar findings, where glycerol inclusion improved breast muscle deposition during the starter phase but showed diminishing effects at later ages, likely due to reduced efficiency of nutrient partitioning as birds approached market weight. Conversely, the improvement in thigh yield during the finisher phase may reflect a shift toward leg muscle and fat deposition at later ages, when breast muscle growth slows. This pattern is consistent with de Castro Tavernari et al [4], who observed that later glycerol supplementation favored leg tissue development over breast accretion.

#### Inclusion level

Inclusion level was another critical factor determining growth performance outcomes. Low-to-moderate supplementation levels (3% to 7%) enhanced BWG, stimulated FI, and improved FCR, while very low levels (1% to 3%) improved efficiency without substantially increasing intake. In contrast, high inclusion levels (>12% to 21%) negatively affected BWG and FCR. These responses may be explained by the balance between CG’s contribution to dietary metabolizable energy at moderate levels and the adverse effects of excessive glycerol, such as increased dietary osmolarity, impaired pellet quality, or residual impurities in CG. Dozier et al [3] reported similar results, noting that moderate glycerol inclusion improved performance, whereas higher levels depressed growth due to reduced feed efficiency. More recently, Carvalho et al [40] confirmed that glycerol can safely replace maize up to 15% of the diet without adverse effects, provided its quality is consistent, thereby supporting the benefits of moderate supplementation as seen in the present analysis.

At low-to-moderate levels (3% to 7%), breast yield was improved, suggesting that glycerol supplied sufficient metabolizable energy to support lean tissue growth without negatively affecting feed quality or digestive physiology. This finding aligns with Broch et al [37], who reported that moderate glycerol inclusion improved breast yield while maintaining carcass proportions. However, at higher inclusion levels (>12%), carcass and breast yields declined, while thigh yield increased. These outcomes may be linked to excessive dietary glycerol, increasing dietary osmolarity, impairing gut function, or introducing residual impurities (methanol, salts) that reduce nutrient utilization efficiency, thereby shifting energy deposition toward fat and leg tissues rather than breast accretion. Dozier et al [3] highlighted similar dose-dependent effects, showing that high glycerol inclusion impaired feed efficiency and consequently altered carcass composition. de Souza et al [22] further demonstrated that dietary glycerol can effectively substitute for conventional cereal grains, such as maize, when of adequate quality, without compromising broiler performance. However, their study also highlighted variability at higher inclusion levels, emphasizing the inclusion-dependent nature of glycerol utilization observed in the present meta-analysis.

## CONCLUSION

This meta-analysis shows that CG is a viable alternative energy source in broiler diets. The CG supplementation improved BWG and feed efficiency without affecting FI, while carcass yields were largely unchanged. However, CG reduced meat pH and ash content and increased drip loss, suggesting potential effects on water-holding capacity. Blood biochemical responses reflected adaptive metabolic adjustments rather than pathological alterations, although interpretation is limited by the inconsistent reporting of advanced liver health markers and histopathological assessments across studies, highlighting the need for future research incorporating these endpoints to better elucidate hepatic responses to CG inclusion. Overall, CG can partially replace corn in broiler nutrition when included at low to moderate levels, and its dietary inclusion should not exceed 7% to minimize negative effects on meat quality.

## Figures and Tables

**Table 1:** Overview of studies used for the meta-analysis

References	Country	Continent	NBT	NT	Explanatory variables				Outcomes

					Strain	Sex	IL	Age^1)^	
[14]	USA	North America	1,440	3	Cobb 500	Male	0–10	1–42	1, 2, 3, 4, 5, 6, 7, 8
[55]	USA	North America	480	3	Cobb 500	Male	0–5	1–42	1, 2, 3, 4, 5, 6, 7, 8
[57]	Greece	Europe	1,920	4	Cobb 500	Male	0–7.5	1–42	1, 2, 3
[17]	Turkey	Europe	270	3	Ross 308	Male	0–10	1–42	1, 2, 3, 4, 10, 11, 12, 13, 14, 15
[19]	Brazil	South America	1,575	5	Cobb 500	Male	0–10	1–42	1, 2, 3, 4, 5, 6, 7, 10
[58]	Turkey	Europe	270	3	Ross 308	Male	0–10	1–42	1, 2, 3, 4, 10, 11, 12, 13, 14, 15, 19
[36]	Turkey	Europe	360	3	Ross 308	Mixed	0–8	1–42	1, 2, 3, 4, 11, 12, 13, 14, 16, 17, 18
[15]	Spain	Europe	630	5	Ross 308	Mixed	0–10	1–21	1, 2, 3, 11, 24, 25, 26
[35]	Brazil	South America	1,350	5	Cobb 500	Male	0–12	1–42	1, 2, 3, 20, 21, 22, 23, 24, 25, 26
[59]	Greece	Europe	400	4	Cobb 500	Male	0–21	1–42	1, 2, 3
[1]	Brazil	South America	1,610	3	Cobb 500	Mixed	0–10	1–42	1, 2, 3, 4, 5, 6, 7, 8, 9, 10
[60]	Brazil	South America	80	4	Cobb 500	Male	0–6	22–42	1, 2, 3, 4, 5, 6, 7, 16, 17, 18, 19, 24, 25, 26
[61]	Brazil	South America	90	3	Cobb 500	Male	0–12	8–42	1, 2, 3
[21]	Thailand	Asia	400	5	Cobb 500	Male	0–10	1–42	1, 2, 3, 4, 5, 6, 7, 9, 10, 21, 22, 23, 24, 25, 26
[37]	Brazil	South America	960	6	Cobb 500	Mixed	0–15	21–42	1, 2, 3, 4, 5, 6, 7, 8, 11
[62]	Thailand	Asia	180	3	Ross 308	Male	0–5	1–42	4, 5, 6, 7, 8, 10, 11, 12, 13, 16, 17, 18, 19, 20, 21, 22, 23, 24, 25, 26
[9]	Iraq	Asia	240	5	Ross 308	Male	0–10	1–42	1, 2, 3, 4, 10, 11, 12, 13, 14, 15, 27, 28, 29, 30, 32, 33, 34, 35, 36, 37, 38
[63]	Canada	North America	720	2	Ross 308	Mixed	0–8	1–35	1, 2, 3
[64]	Brazil	South America	540	2	Cobb 500	Male	0–7	1–42	1, 2, 3, 4, 5, 6, 7, 8, 10, 11, 13
[42]	Brazil	South America	2,400	5	Cobb 500	Male	0–8	1–42	1, 2, 3, 4, 5, 6, 7, 8, 10, 11, 13
[39]	Brazil	South America	240	4	Cobb 500	Male	0–6	1–42	1, 2, 3, 4, 5, 6, 7, 11, 12, 13, 16, 17, 18, 19, 20, 21, 22, 23, 24, 25, 26
[22]	Brazil	South America	1,056	6	Cobb 500	Male	0–9	1–42	1, 2, 3, 4, 5, 6, 7, 8, 10, 17, 18, 20, 21, 23, 24, 25, 26, 27, 28, 30, 31, 34, 35
[46]	India	Asia	300	3	Cobb 500	Mixed	0–6	1–42	4, 5, 6, 12, 15, 27, 28, 29, 30, 32, 33, 37, 38
[4]	Brazil	South America	1,600	4	Cobb 500	Male	0–12	1–42	1, 2, 3, 4, 5, 6, 7, 8, 9, 10, 11, 12, 13
[40]	Brazil	South America	400	4	Cobb 500	Male	0–15	1–42	1, 2, 3, 4, 5, 6, 7, 10, 11, 12, 14, 27, 28, 29

1 = body weight gain, 2 = feed intake, 3 = feed conversion ratio, 4 = carcass yield, 5 = breast yield, 6 = thigh yield, 7 = drumstick, 8 = wings, 9 = back yield, 10 = abdominal fat, 11 = liver, 12 = gizzard, 13 = heart, 14 = proventriculus, 15 = spleen, 16 = ash, 17 = ether extract, 18 = crude protein, 19 = moisture, 20 = pH, 21 = cook loss, 22 = drip loss, 23 = shear force, 24 = L*, 25 = a*, 26 = b*, 27 = cholesterol, 28 = triglycerides, 29 = total protein, 30 = glucose, 31 = creatinine, 32 = albumin, 33 = globulin, 34 = aspartate aminotransferase (AST), 35 = alanine aminotransferase (ALT), 36 = alkaline phosphatase (ALP), 37 = high-density lipoprotein (HDL), 38 = low-density lipoprotein (LDL).

1) In days.

NBT, number of birds in the study; NT, number of treatments; IL, inclusion level (%).

**Table 2:** Growth performance indices of broiler chickens fed crude glycerol

Outcomes	NC	Estimate (SMD)	95 % CI		Std. error	p-value	Heterogeneity				
	
			Lower bound	Upper bound			τ^2^	Q	df	Het. p-value	I^2^
Growth performance											
BWG	170	0.398	0.203	0.592	0.099	<0.001	1.203	655.684	169	<0.001	74.225
FI	170	0.061	−0.109	0.232	0.087	0.481	0.850	530.556	169	<0.001	68.147
FCR	168	−0.444	−0.616	−0.272	0.088	<0.001	0.778	453.841	167	<0.001	65.406

NC, number of comparisons; SMD, standardized mean difference; CI, confidence interval; τ^2^, measure of heterogeneity; Q, Cochran statistic; df, degree of freedom; I^2^, inconsistency index; BWG, body weight gain; FI, feed intake; FCR, feed conversion ratio.

**Table 3:** Carcass traits and internal organ weights of broiler chickens fed crude glycerol

Outcomes	NC	Estimate (SMD)	95% CI		Std. error	p-v alue	Heterogeneity				
	
			Lower bound	Upper bound			τ^2^	Q	df	Het. p-value	I^2^
Carcass traits											
Carcass yield	60	−0.037	−0.238	0.164	0.103	0.719	0.223	92.307	59	0.004	36.083
Breast	50	0.307	0.116	0.498	0.097	0.002	0.091	60.793	49	0.120	19.398
Thigh	46	0.275	−0.037	0.588	0.159	0.084	0.741	129.616	45	<0.001	65.282
Drumstick	41	0.350	0.016	0.683	0.170	0.040	0.771	121.071	40	<0.001	66.961
Wings	34	−0.059	−0.294	0.175	0.120	0.619	0.131	45.439	33	0.073	27.376
Back	18	0.769	0.283	1.254	0.247	0.002	0.680	47.259	17	<0.001	64.028
Abdominal fat	32	−0.368	−0.846	0.109	0.243	0.130	1.371	123.374	31	<0.001	74.873
Internal organs											
Liver	35	0.387	0.008	0.766	0.193	0.045	0.869	107.526	34	<0.001	68.38
Gizzard	21	−0.625	−1.061	−0.188	0.223	0.005	0.579	45.838	20	<0.001	56.369
Heart	23	0.255	−0.214	0.724	0.239	0.286	0.847	63.765	22	<0.001	65.498
Proventriculus	13	−0.594	−1.010	−0.178	0.212	0.005	0.095	14.339	12	0.280	16.313
Pancreas	4	0.034	−0.659	0.727	0.354	0.923	0.000	0.040	3	0.9980	0
Spleen	10	−0.248	−0.673	0.177	0.217	0.253	0.000	4.373	9	0.885	0

NC, number of comparisons; SMD, standardized mean difference; CI, confidence interval; τ^2^, measure of heterogeneity; Q, Cochran statistic; df, degree of freedom; I^2^, inconsistency index.

**Table 4:** Meat quality parameters of broiler chickens fed crude glycerol

Outcomes	NC	Estimate (SMD)	95 % CI		Std. error	p-value	Heterogeneity				
	
			Lower bound	Upper bound			τ^2^	Q	df	p-value	I^2^
Chemical composition											
Ash	12	−1.061	−1.648	−0.473	0.300	<0.001	0.531	22.064	11	0.024	50.144
Ether extract	15	0.548	−0.143	1.240	0.353	0.120	1.300	47.043	14	<0.001	70.240
Crude protein	15	0.358	−0.255	0.970	0.313	0.253	0.933	39.230	14	<0.001	64.313
Moisture	13	0.521	−0.044	1.087	0.289	0.071	0.592	27.009	12	0.008	55.570
Physical properties											
pH	22	−0.326	−0.640	−0.012	0.160	0.042	0.097	25.370	21	0.231	17.226
Cook loss	18	−0.270	−0.729	0.189	0.234	0.248	0.482	33.585	17	0.009	49.382
Drip loss	16	0.582	0.185	0.979	0.203	0.004	0.160	19.810	15	0.179	24.280
Shear force	18	0.047	−0.294	0.388	0.174	0.786	0.074	19.701	17	0.290	13.711
Color											
Lightness (L*)	21	0.329	−0.076	0.734	0.207	0.112	0.405	36.683	20	0.013	45.478
Redness (a*)	21	0.041	−0.328	0.410	0.188	0.828	0.265	31.221	20	0.052	35.940
Yellowness (b*)	21	−0.331	−0.996	0.335	0.340	0.330	1.582	77.562	20	<0.001	74.214

NC, number of comparisons; SMD, standardized mean difference; CI, confidence interval; τ^2^, measure of heterogeneity; Q, Cochran statistic; df, degree of freedom; I^2^, inconsistency index.

**Table 5:** Blood biochemical parameters of broiler chickens fed crude glycerol

Outcomes	NC	Estimate (SMD)	95 % CI		Std. error	p-value	Heterogeneity				
	
			Lower bound	Upper bound			τ^2^	Q	df	p-value	I^2^
Blood biochemical											
Cholesterol	14	−0.743	−1.187	−0.299	0.227	0.001	0.246	19.875	13	0.098	34.590
Triglycerides	14	−0.301	−0.666	0.064	0.186	0.106	0.051	14.529	13	0.338	10.524
Total protein	14	0.015	−0.422	0.452	0.223	0.946	0.214	18.811	13	0.129	30.890
Glucose	14	1.184	0.438	1.931	0.381	0.002	1.312	39.867	13	<0.001	67.392
Creatinine	12	1.191	−0.353	2.735	0.788	0.131	4.688	76.902	11	<0.001	85.696
Albumin	12	−1.388	−2.276	−0.501	0.453	0.002	1.761	41.118	11	<0.001	73.247
Globulin	12	−0.031	−0.424	0.363	0.201	0.879	0.006	11.132	11	0.432	1.186
AST	14	0.203	−0.537	0.943	0.378	0.591	1.299	41.917	13	<0.001	68.986
ALT	14	−1.000	−1.709	−0.291	0.362	0.006	1.156	37.662	13	<0.001	65.483
ALP	14	0.530	0.049	1.010	0.245	0.031	0.298	20.230	13	0.090	35.739
HDL	12	0.665	0.256	1.073	0.208	0.001	0.016	11.346	11	0.415	3.053
LDL	12	−1.317	−1.770	−0.864	0.231	<0.001	0.057	12.070	11	0.358	8.863

NC, number of comparisons; SMD, standardized mean difference; CI, confidence interval; τ^2^, measure of heterogeneity; Q, Cochran statistic; df, degree of freedom; I^2^, inconsistency index; AST, aspartate aminotransferase; ALT, alanine aminotransferase; ALP, Alkaline phosphatase; HDL, high-density lipoprotein; LDL, low-density lipoprotein.

**Table 6:** Subgroup analyses of the effect of covariates of CG on BWG, FI, and FCR of broiler chickens

Outcomes	Moderators	Subgroup	NC	Estimate (SMD)	Lower bound	Upper bound	Std. error	p-value
BWG	Strain	Cobb 500	124	0.207	−0.028	0.442	0.120	0.084
		Ross 308	46	0.905	0.620	1.190	0.145	<0.001
	Sex	Male	149	0.345	0.135	0.556	0.108	0.001
		Mixed	21	0.747	0.246	1.248	0.256	0.003
	Rearing phases	Starter (1–14)	60	0.571	0.215	0.928	0.182	0.002
		Grower (14–28)	19	0.808	0.199	1.417	0.311	0.009
		Finisher (28–42)	39	0.021	−0.285	0.327	0.156	0.893
		Overall (1–42)	52	0.380	0.008	0.752	0.190	0.046
	Inclusion levels (%)	Very low (1–3)	27	0.200	−0.046	0.445	0.125	0.112
		Low to moderate (>3–7)	68	0.973	0.680	1.266	0.150	<0.001
		Moderate to high (>7–12)	62	0.075	−0.288	0.439	0.186	0.685
		High inclusion (>12–21)	13	−0.747	−1.395	−0.099	0.331	0.024
FI	Strain	Cobb 500	124	−0.366	−0.561	−0.172	0.099	<0.001
		Ross 308	46	−0.663	−1.028	−0.298	0.186	<0.001
	Sex	Male	149	−0.447	−0.618	−0.276	0.087	<0.001
		Mixed	21	−0.471	−1.128	0.186	0.335	0.160
	Rearing phases	Starter (1–14)	60	0.117	−0.134	0.369	0.128	0.360
		Grower (14–28)	19	0.504	0.036	0.972	0.239	0.035
		Finisher (28–42)	39	−0.124	−0.499	0.251	0.191	0.517
		Overall (1–42)	52	−0.015	−0.361	0.331	0.177	0.932
	Inclusion levels (%)	Very low (1–3)	27	−0.103	−0.353	0.146	0.127	0.417
		Low to moderate (>3–7)	68	0.507	0.287	0.727	0.112	<0.001
		Moderate to high (>7–12)	62	−0.338	−0.695	0.018	0.182	0.062
		High inclusion (>12–21)	13	−0.338	−0.939	0.263	0.307	0.271
FCR	Strain	Cobb 500	112	−0.123	−0.336	0.090	0.109	0.259
		Ross 308	46	0.540	0.345	0.736	0.100	<0.001
	Sex	Male	137	0.043	−0.145	0.232	0.096	0.653
		Mixed	21	0.195	−0.187	0.576	0.194	0.317
	Rearing phases	Starter (1–14)	60	−0.673	−0.967	−0.379	0.150	<0.001
		Grower (14–28)	15	−0.154	−0.659	0.352	0.258	0.551
		Finisher (28–42)	35	−0.094	−0.442	0.255	0.178	0.598
		Overall (1–42)	48	−0.517	−0.822	−0.213	0.155	<0.001
	Inclusion levels (%)	Very low (1–3)	23	−0.326	−0.580	−0.071	0.130	0.012
		Low to moderate (>3–7)	64	−0.716	−0.960	−0.472	0.124	<0.001
		Moderate to high (>7–12)	58	−0.423	−0.766	−0.079	0.175	0.016
		High inclusion (>12–21)	13	0.554	0.112	0.997	0.226	0.014

CG, crude glycerol; BWG, body weight gain; FI, feed intake; FCR, feed conversion ratio; NC, number of comparisons; SMD, standardized mean difference.

**Table 7:** Subgroup analyses of the effect of covariates of CG on carcass yield, breast, and thigh of broiler chickens

Outcomes	Moderators	Subgroup	NC	Estimate (SMD)	Lower bound	Upper bound	Std. error	p-value
Carcass yield	Strain	Cobb 500	46	0.005	−0.208	0.219	0.109	0.961
		Ross 308	12	−0.295	−0.923	0.333	0.320	0.357
	Sex	Male	49	−0.058	−0.309	0.193	0.128	0.652
		Mixed	11	0.058	−0.274	0.390	0.170	0.732
	Rearing phases	Starter (21)	5	1.087	−0.035	2.208	0.572	0.058
		Finisher (42)	55	−0.100	−0.293	0.094	0.099	0.313
	Inclusion levels (%)	Very low (1–3)	13	0.171	−0.175	0.517	0.177	0.333
		Low to moderate (>3–7)	25	0.046	−0.228	0.320	0.140	0.743
		Moderate to high (>7–12)	20	−0.373	−0.807	0.062	0.222	0.093
		High inclusion (>12–21)	2	0.313	−0.698	1.324	0.516	0.544
Breast	Strain	Cobb 500	46	0.302	0.099	0.505	0.104	0.004
		Ross 308	2	0.042	−0.940	1.024	0.501	0.933
	Sex	Male	41	0.333	0.098	0.568	0.120	0.005
		Mixed	7	0.214	−0.141	0.569	0.181	0.237
	Rearing phases	Starter (21)	5	1.161	0.254	2.068	0.463	0.012
		Finisher (42)	45	0.251	0.065	0.437	0.095	0.008
	Inclusion levels (%)	Very low (1–3)	12	0.279	−0.124	0.682	0.205	0.175
		Low to moderate (>3–7)	21	0.326	0.063	0.589	0.134	0.015
		Moderate to high (>7–12)	15	0.338	−0.095	0.771	0.221	0.126
		High inclusion (>12–21)	2	−0.106	−0.880	0.668	0.395	0.788
Thigh	Strain	Cobb 500	40	0.296	−0.057	0.649	0.180	0.100
		Ross 308	6	0.176	−0.393	0.745	0.290	0.545
	Sex	Male	39	0.295	−0.085	0.676	0.194	0.129
		Mixed	7	0.204	−0.177	0.585	0.194	0.295
	Rearing phases	Starter (21)	5	−0.860	−1.803	0.082	0.481	0.074
		Finisher (42)	41	0.392	0.072	0.712	0.163	0.016
	Inclusion levels (%)	Very low (1–3)	11	0.189	−0.413	0.792	0.307	0.538
		Low to moderate (>3–7)	19	0.031	−0.427	0.489	0.234	0.895
		Moderate to high (>7–12)	14	0.573	−0.086	1.232	0.336	0.088
		High inclusion (>12–21)	2	1.091	0.259	1.922	0.424	0.010

CG, crude glycerol; NC, number of comparisons; SMD, standardized mean difference.

## Data Availability

Upon reasonable request, the datasets of this study can be available from the corresponding author.

## SUPPLEMENTARY MATERIAL

**Supplement 1**. PRISMA 2020 Checklist for the meta-analysis of dietary crude glycerol in broiler chickens.
