# Supplementary material for: Dietary crude glycerol as an energy source in broiler chickens: a meta-analysis
Source: Anim Biosci. 2026 Mar 11;39(7):250686. doi: 10.5713/ab.250686 (PMC13353112; doi:10.5713/ab.250686)
Supplement: Supplementary file 1 [file ab-250686-Supplementary-1.pdf]

Supplement 1. PRISMA 2020 Checklist for the meta-analysis of dietary crude glycerol in broiler chickens

Section/Topic	Item No.	Checklist description	Location in manuscript
TITLE	1	Identify the report as a systematic review and meta-analysis	Title page, line 1-2
ABSTRACT	2	Structured summary of objectives, eligibility, sources, methods, results, and conclusions.	Abstract section p. 3
INTRODUCTION	3	Rationale: Describe the rationale for the review in the context of existing knowledge	Introduction, first three paragraphs, p. 4-5
	4	Objectives: Provide an explicit statement of the objectives/questions addressed	Introduction, last paragraph, p. 5
METHODS	5	Eligibility criteria: Specify inclusion and exclusion criteria, including outcomes for the review	Materials and Methods: Inclusion and Exclusion Criteria, p. 6-7
	6	Information sources: Describe all information sources (databases, search, and dates)	Materials and Methods: Literature Search procedure, p. 6
	7	Search strategy: Present full search terms, strategy with limits used.	Materials and Methods: Literature Search, p. 6
	8	Selection process: State process for selecting studies (screening, eligibility, inclusion)	Materials and Methods: Literature Search & Inclusion Criteria, p. 6-7
	9	Data collection process: Describe how the data were extracted from reports	Materials and Methods: Data Extraction, p. 7
	10	Data items: List and	Materials and

		define variables for which data were sought	Methods: Data Extraction, p. 7
	11	Study risk of bias assessment: Specify methods used to assess bias.	Not performed (limitation noted in Discussion)
	12	Effect measures: Specify effect measures used for each outcome	Materials and Methods: Data Analysis, p. 7-8
	13	Synthesis methods: Describe how studies were synthesized	Materials and Methods: Data Analysis, p. 7-8
	14	Heterogeneity assessment: Methods used to explore causes of heterogeneity	Materials and Methods: Subgroup and sensitivity analyses, p. 8
	15	Subgroup and sensitivity analyses performed.	Materials and Methods: Subgroup and sensitivity analyses, p. 8
	16	Publication bias assessment	Materials and Methods: Publication bias evaluation (funnel plot, Egger's test), p. 8
Results	17	Study selection: Report numbers screened, assessed, and included, with reasons for exclusion	Results: Study selection; Figure 1 (PRISMA flow diagram),
	18	Study characteristics: Present characteristics for each study	Results: Tables 1 (studies included) p. 32
	19	Risk of bias in studies	Not assessed explicitly (acknowledged in Discussion)
	20	Results of individual studies: Provide summary data for each study	Results: Tables 2–7, Figures 1-2
	21	Results of syntheses: Summarize effect estimates and confidence intervals	Results: Growth Performance, Carcass Traits, Meat Quality, Blood Biochemistry, p. 33-38

	22	Heterogeneity, sensitivity analyses, and subgroup	Results: p. 37–38
Discussion	23	Summary of main findings	Discussion, p. 13–22
	24	Limitations of evidence and review process	Discussion – Limitations, p. 22
	25	Implications for practice, policy, and research	Discussion – p. 13–22; Conclusion, p. 22
Other	26	Registration and protocol	Not registered (stated in Methods/PRISMA statement)
	27	Support: Describe funding sources	Funding section, p. 23
	28	Competing interests	Conflict of Interest statement, p. 23
